# Supplementary material for: Preparing for Practice: An Exploration of Health and Social Care Professionals’ Perceptions of Behaviour Change Education
Source: Behav Sci (Basel). 2025 Nov 8;15(11):1523. doi: 10.3390/bs15111523 (PMC12649299; doi:10.3390/bs15111523)
Supplement: Supplementary file 1 [file behavsci-15-01523-s001.zip › behavsci-3920510-supplementary.pdf]

Supplementary Material S1

Survey measures presented in the manuscript

Course Characteristics

We are interested in understanding your perceptions and experiences of health behaviour change education during your course.

Health behaviour change can be defined as: *"Prompting changes or influencing the factors that lead to changes in health behaviour, to help individuals develop healthy habits and make positive changes in their everyday behaviours."*

Below are some examples of health behaviour change:

Behaviour change theories or models	Stages of Change, Social Cognitive Theory, The Transtheoretical Model/Stages of Change, the Health Belief Model, and the Theory of Planned Behaviour
Counselling techniques	Motivational interviewing, cognitive behavioural therapy, acceptance commitment therapy
Communication strategies	Active listening, summarising, affirmations
Behaviour change techniques	Problem solving, reattribution, goal setting, self-monitoring, information about consequences
Using interventions designed to change behaviour beyond individual settings	Group education, adherence strategies

Behaviour change – Focus

Thinking about your course, how much **focus** did your course place on the following health behaviour change concepts:	None at all	A little	A moderate amount	A lot	A great deal
Behaviour change theories or models and how to implement them (e.g., stages of change)	○	○	○	○	○
Counselling therapies (e.g., motivational interviewing, cognitive behavioural therapy)	○	○	○	○	○
Communication skills (e.g., active listening, affirmations)	○	○	○	○	○
Behaviour change techniques (e.g., goal setting, pros and cons, reframing, habit formation)	○	○	○	○	○
Using interventions designed to change behaviour beyond individual settings (e.g., group education, adherence strategies)	○	○	○	○	○

Behaviour change - Integration

Thinking about your course, to what extent do you feel that health behaviour change training was **integrated** across the course? (i.e., building your skills across unit/topics and year level).

	None at all	A little	A moderate amount	A lot	A great deal
Behaviour change theories or models and how to implement them	○	○	○	○	○
Counselling therapies (e.g., motivational interviewing)	○	○	○	○	○
Communication skills (e.g., active listening, affirmations)	○	○	○	○	○
Behaviour change techniques (e.g., goal setting, pros and cons, reframing, habit formation)	○	○	○	○	○
Using interventions designed to change behaviour beyond individual settings (e.g., group education, adherence strategies)	○	○	○	○	○

Behaviour change - Inclusion

Thinking about your course, how much did your course **include** health behaviour change training in the following areas:

	None at all	A little	A moderate amount	A lot	A great deal
Lectures (e.g., theory coverage)	○	○	○	○	○
Workshops & Tutorials (e.g., where there are practical opportunities & role plays)	○	○	○	○	○
Assessments (e.g., practical assessments, OSCEs, written, oral presentations, group work)	○	○	○	○	○
Placements (e.g., community, hospital)	○	○	○	○	○

Perceived knowledge

On a scale of (1) *no understanding* to (7) *full understanding*, how would you rate your **knowledge** of health behaviour change following your course?

Please slide the bar to select your response (if your response is 0, please just click the bar)

	No understanding				Full understanding		
	0	1	2	4	5	6	7
Behaviour change theories or models and how to implement them							
Counselling therapies (e.g., motivational interviewing)							
Communication skills (e.g., active listening, affirmations)							
Behaviour change techniques (e.g., goal setting, pros and cons, reframing, habit formation)							
Using interventions designed to change behaviour beyond individual settings (e.g., group nutrition education, dietary adherence strategies)							

Satisfaction

Thinking about your course, how **satisfied** are you with how the components of the course have prepared you to deliver health behaviour change in your current/future role?

	Extremely dissatisfied	Somewhat dissatisfied	Neither satisfied nor dissatisfied	Somewhat satisfied	Extremely satisfied
Inclusion of health behaviour change content (e.g, theories, techniques) in the course	○	○	○	○	○
Teaching quality of health behaviour change	○	○	○	○	○
Skills learned to change behaviour (e.g., counselling strategies techniques)	○	○	○	○	○
Opportunities to practice skills in health behaviour change (e.g., OSCEs, placements)	○	○	○	○	○
Overall course	○	○	○	○	○

Confidence, perceived importance, and perceived usefulness

The following questions ask about your **confidence, perceived importance, and usefulness** of having behaviour change conversations with patients after completing your course. Please respond to each statement from *'not at all'* to *'extremely*.'

	Not at all	Slightly	Moderately	Very	Extremely
How **confident** do you feel about supporting patients to make behaviour changes?	○	○	○	○	○
How **important** is it for you to support patients to make behaviour changes?	○	○	○	○	○
How **useful** do you think the conversations you have are helping support patients to make behaviour changes?	○	○	○	○	○

Theoretical Domains Framework

Please reflect on the following statements and questions about **having behaviour change conversations with patients** based on the knowledge and skills you have received from your course. Please indicate how much you agree with each of the following statements from *'strongly disagree'* to *'strongly agree'*.

Note: A 'behaviour change conversation' is a talk or discussion with patients about changing their actions or habits to improve their health and wellbeing. It could include topics such as healthy eating, physical activity, treatment or medication adherence, hydration and reducing alcohol intake.

	Strongly disagree	Disagree	Somewhat disagree	Neutral	Somewhat agree	Agree	Strongly agree
I have **been trained in how** to have behaviour change conversations with patients.	○	○	○	○	○	○	○
I have **the skills** to have behaviour change conversations with patients.	○	○	○	○	○	○	○
I have **practiced** having behaviour change conversations with others since completing my course.	○	○	○	○	○	○	○
Having behaviour change conversations with patients is **part of my work** as a health professional.	○	○	○	○	○	○	○
It is **my responsibility** as a health professional to have behaviour change conversations with patients.	○	○	○	○	○	○	○
If I have behaviour change conversations in routine consultations with patients, **it will benefit public health.**	○	○	○	○	○	○	○
I am confident that I can have behaviour change conversations with patients **even when patients are not motivated.**	○	○	○	○	○	○	○
I am confident that I can have behaviour change conversations with patients **even when there is little time.**	○	○	○	○	○	○	○
If I have behaviour change conversations with patients, **it will have disadvantages for my relationship with patients.**	○	○	○	○	○	○	○
**I will definitely** have behaviour change conversations with patients in the next 3 months.	○	○	○	○	○	○	○
I **intend** to have behaviour change conversations with patients **in the next 3 months.**	○	○	○	○	○	○	○
Behaviour change conversations with patients is **something I do without thinking.**	○	○	○	○	○	○	○

Behaviour [Only provided to Phase 2 sample]

In a typical week, how **often** do you have behaviour change conversations with patients or clients (e.g., about diet, physical activity, smoking, alcohol, or medication adherence)?

- ◯ None of the time
- ◯ Rarely
- ◯ Some of the time
- ◯ Most of the time
- ◯ All of the time

Applicability

The next few questions will ask you about how applicable and translatable the skills from your course were for your current role. After completing your course, how **applicable** do you feel that the health behaviour change knowledge and skills acquired during your course are to your current or future professional role?

- ◯ Not applicable at all
- ◯ Somewhat applicable
- ◯ Moderately applicable
- ◯ Very applicable
- ◯ Extremely applicable

Upskill

Following your course, how much do you feel you have (or had to) upskill your health behaviour change skills to work with patients?

- ◯ Not at all
- ◯ A little
- ◯ A moderate amount
- ◯ A lot
- ◯ A great deal

CPD

How likely will you be to **seek professional development** in behaviour change techniques and approaches now your course has finished?

- ○ Extremely unlikely
- ○ Somewhat unlikely
- ○ Neither likely nor unlikely
- ○ Somewhat likely
- ○ Extremely likely
- ○ I have already completed professional development in this area.

***Please note. Items included in Phase 1 were varied slightly in item wording to reflect nutrition and dietetics and pharmacy contexts.*
